# Analysis of microplastics in the reuse of compost in three agricultural sites (Cádiz, Spain) as a circular economy strategy: detection of micropollutants and incidence of plastic ingestion levels by annelids

**DOI:** 10.1007/s11356-024-34615-w

**Published:** 2024-08-10

**Authors:** Ayda Sakali, Agata Egea-Corbacho, Dolores Coello, Gemma Albendín, Juana Arellano, Rocío Rodríguez-Barroso

**Affiliations:** 1 Department of Environmental Technologies, Faculty of Marine and Environmental Sciences, INMAR-Marine Research Institute, CEIMAR International Campus of Excellence of the Sea, University of Cadiz, Campus Universitario de Puerto Real, 11510 Cadiz, Spain; 2 Toxicology Department, International Campus of Excellence of the Sea (CEIMAR), Faculty of Marine and Environmental Sciences, University Institute of Marine Research (INMAR), University of Cádiz, 11510 Puerto Real, Spain

**Keywords:** Microplastics, Compost, Southern Spain, Soil, Annelids, PTFE

## Abstract

The system of fertilizing agricultural soils with sludge or compost from wastewater treatment processes, as one of the principles of the circular economy, can lead to microplastic (MP) contamination. The existing technical standards for fertilization are very recent and do not consider this problem, although there is scientific evidence of their existence. Therefore, this study, on the one hand, evaluates the presence of MPs in agricultural soils, previously treated with sludge or compost from wastewater treatment plants for fertilization, and on the second hand, it studies the effect of these MPs on earthworms in three different locations in the south of Spain. For the study, selected composts deriving from the different stages of the composting process and three fertilized soils with increasing MP doses were followed. Samples were taken from different sections in depth (0–5, 5–10, and 10–20 cm) to study the shape, size, type, and abundance of MPs using infrared spectroscopy (FTIR). The results showed that the most abundant shape was fiber, followed by fragment and finally bulk, for both composts and soils. Regarding size distribution, 100 µm was the predominant size in composts (64.3% ± 9.8), while in the case of soils, the predominant range was from 100 to 500 µm. The prevalent polymers in both, composts and soils, were PTFE, TPE, PP, and PET, with four times higher amounts in composts than in soils. Ingestion of common MPs were also verified in two earthworm species, which ingested concentrations higher than 2.1% w/w. PP was the most ingested MP and *Eisenia fetida* was more voracious compared with *Lumbricus terrestris*. Therefore, it can be considered a suitable bioindicator for monitoring microplastic contamination in agricultural soil.

**Graphical Abstract:**

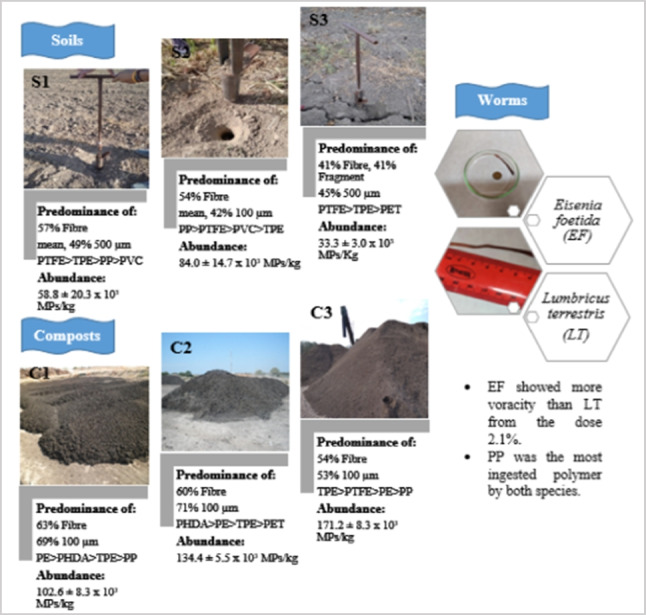

## Introduction

Plastic container waste has increased rapidly with home learning and teleworking caused by COVID-19 (Haddad et al. 2021). Plastics have proven to be a key weapon in this fight. Degraded physically, chemically, and biologically, they create small debris, called microplastics (MPs), a catch-all term to describe any water-insoluble solid plastic particle between 1 and 1000 µm in size (ISO 2020). MPs do not fall directly within the scope of the directive (EU) 2019/904 of the European Parliament and the Council on the reduction of certain plastic products’ impact on the environment. However, they contribute to marine litter; therefore, the European Union should therefore adopt a comprehensive approach applying to MPs.

MPs can be transported from both ocean and land, but their behavior in the soil is still not well-known. Terrestrial pollution by polymer items can be significant, on account of their high specific surface area as well as their physicochemical properties. Moreover, MPs are considered to be vectors for other soil contaminants, such as potentially toxic elements (PTEs) and organic contaminants (Igalavithana et al. 2022). Several studies mention different toxic compounds that can be adsorbed by MPs (Bradney et al. 2019; Thacharodi et al. 2024), like PAHs, PCBs (Zhang & Chen 2020; Xie et al. 2024), additives (Edo et al. 2020; Li et al. 2024), metals (Li et al. 2021), and neonics (Gross et al. 2022). Therefore, the combination of MPs including PTEs can help spread toxic effects from one environment to another (Tadsuwan & Babel 2021). The soil as an essential media justifies its exploration considering that the occurrence of MPs can change the living environment of soil fauna, such as earthworms (Zhang et al. 2022; Tian et al. 2024), and threaten soil properties (Chia et al. 2022). Soil organic carbon and total nitrogen stock are called into question since the additional greenhouse effect coupled with radiative forcing rise the Earth’s energy budget, which is dissipated into the ground and warms up the atmosphere, leading to heatwaves, organic matter relinquishment, and permafrost crushing, encouraging the outdoor MP infiltration in the indoor environment (Wang et al. 2019).

Wastewater discharges from wastewater treatment plants (WWTP) are one of the main sources of microplastics from urban agglomerations followed by atmospheric deposition and surface runoff (Zhou et al. 2023). Thus, sewage systems and networks represent an important source of polymers that transport MPs to WWTPs. In sewer systems, MPs can undergo degradation (biotic and abiotic), adsorption, and retransformation, but they are not removed in either sewer networks or WWTPs (Compagni, et al. 2019). WWTPs have preliminary, primary, and secondary treatments that are not selective or specific for the removal of MPs and therefore do not remove them but can retain and concentrate them in the sludge line (Egea-Corbacho et al. 2023; Dronjak et al. 2023). Preliminary screens and grit chambers remove large debris, gravel, and sand. Primary treatment removes grease and settleable particles (El Mansouri et al. 2022). Secondary treatments in WWTPs include both aerobic and anaerobic processes, within the anaerobic ones, and dissolved organic matter is degraded into carbon dioxide and water (Bilgin et al. 2020) through bioremediation generating gasses such as methane (Masiá et al. 2020; Huang et al. 2024). Wu et al. (2020) showed that MP levels might inhibit methane production compared with the negative controls. Another study showed the importance of MP size, as nanometer-sized MPs inhibited sludge activity and decreased the abundance of key microorganisms, which subsequently altered the composition and spatial distribution of extracellular polymeric substances (EPS) and ultimately prevented sludge dewatering (Xu et al. 2021).

The potential for MPs bioremediation in WWTPs is more evident in the gravitational settling of the sewage sludge. Therefore, the size, toxicity, and type of polymers in the sludge must be taken into account, because its accumulation is not risk-free. Sludge is reused in agriculture with the aim of valorizing a residue according to the principles of the circular economy (efficiency in the sustainable use of resources), in addition to the fact that it is beneficial to agriculture; actions aligned with SDGs 14 (Life on land) and SDGs 12 (Responsible production and consumption). Thus, the appropriate management of sludge should be considered important to prevent the MP release from WWTPs.

It is evident that environmental conditions, soil attributes, vegetative cowl, and temporal arrangement of uses sway microplastic soil maintenance (Crossman et al. 2020; Torres et al. 2021). Recent studies related to the identification of MPs in soil revealed that the stabilization of wastewater solids, creating end products such as conventional compost (El Hayany et al. 2020; Vo et al. 2024) or simply dewatered sludge (Li et al. 2019), leads to their use as fertilizers (Corradini et al. 2019; Zhang et al. 2020), and despite efforts to dispose of MPs from WWTPs, a considered percentage still bypasses the removal stage and translocate to soil (Bradney et al. 2019; Long et al. 2024), from where it is estimated that in Europe alone, 125–850 tons of MPs/million inhabitants a year are added to agricultural soils via sewage sludge applications (Bank 2022; Mai et al. 2024). Furthermore, it has been calculated that the average amount of MPs derived from sewage sludge that can enter the Spanish soil is 2.80 × 10^14^ MPs/year (Sakali et al. 2022).

Deteriorative reactions occur during processing when polymers, often compared with sediments in a simplified manner (Waldschläger 2020), are subject to the most important degradative organisms. Forest canopies (Murazzi et al. 2022) and pine needles (Liu et al. 2022) are also affected and evaluated as good receptors of MPs atmospheric deposition. Earthworms can eventually deteriorate the natural balance of agricultural systems as they are involved in important ecological processes such as ammonization, nitrification and denitrification (Shen et al. 2022).

The unpredictable presence of harmful polymers that persist in terrestrial ecosystems, as well as their capacity to absorb pollutants and the difficulties in detecting them, can generate potential effects on soil and organisms, which is the focus of this study. Therefore, in order to prevent MPs from entering the ecosystem, more specifically the soil, and generating the problems mentioned above, more studies are required to understand their inclusion arrival and behavior in the environment. In addition, these studies will allow the development of stricter standards for both agriculture and sludge management, additionally to the improvement of technologies and management strategies, which results in a great challenge for industry and research (Ivleva 2021; van den Berg et al. 2020; Meng et al. 2020).

This study is aimed at investigating the presence of MPs in agricultural soils that have been previously fertilized with sludge or compost coming from WWTPs. In addition, the ingestion capacity of earthworms will also be evaluated as bioindicators. For this purpose, the composition, diversity, and abundance of MPs in three composts derived from conventionally treated sludge throughout the composting process as well as in three sludge-enriched soils were investigated. Also, two types of earthworms were studied as possible bioindicators to check the level of ingestion at increasing doses of the most common MPs found (0.9, 2.1 and 3% w/w).

To monitor MP levels, we developed a method for the MPs analysis, by using infrared spectroscopy (FTIR) to identify polymer features and provided evidence to confirm that sludge and compost are relevant land-based sources of MPs in the agrosystems. It is hypothesized that the abundance and size of MPs in agricultural soils may increase with increasing fertilization and composting stage.

## Materials and methods

### Sample collection

The analyzed compost comes from a plant located in Southern Spain, owned by the company Valoriza Medioambiente (SACYR). It combines sewage sludge and vegetable biomass from municipal parks and gardens, obtaining as a final product an organic fertilizer with very good agronomic characteristics, mainly suitable for agricultural use.

In general terms, the composting process consists of spreading sewage sludge over drying beds for a few weeks at ambient temperature (humid compost, C1). Then, the sludge is piled up, mixed with other by-products such as manure, rotten straw and pruning for fermentation (semi-dried compost, C2). The last step consists of conditioning and refining the residue by sieving and eliminating the rejects (fine compost, C3). A representative sample (~ 1 kg, mean moisture content 7.48 ± 4.54%) of each compost type was collected and frozen as soil samples until analysis. With the help of a sludge management company in the region (Grupo Valora), two types of sludge-enriched soils and one control soil were selected from different locations in the province of Cádiz.

- S1: a field with less than 2 years of fertilization (at the highest dose 30 t/ha) plowed twice/year (36° 44′ 40.38″ N; − 6° 20′ 57.12″ W).
- S2: a sunflower field with more than 4 years of fertilization (at the lowest dose 15 t/ha) plowed twice/year (36° 36′ 51.372″ N; − 6° 10′ 36.84″ W).
- S3 (Control): a chickpea field without exposure to sludge (36° 23′ 35.16″ N; − 5° 54′ 16.128″ W).

A noteworthy fact is that the study examined the same sludge before being composted and after it had been applied to the soil. S1, S2, and S3 were silty clay to clayey textured soils, which are considered good for agriculture, with no evidence of early antiweed netting or temporary greenhouses.

From each soil, four random sampling points were collected and integrated into a single sample. Using a metal soil auger, S1 and S2 samples were first taken from the topsoil layer (0–5 cm), followed by the middle layer (5–10 cm) and finally the deep layer (10–20 cm). These depths were selected to evaluate the polymer content in the root zone. Previously, each sample (~ 300 g, mean moisture content 0.58 ± 0.37%) was sieved, in situ, through a stainless mesh < 5 mm ø to avoid any possible coarse branches or residues. All soils experienced similar edaphic conditions; the average temperature recorded was 23.44 °C, ranging from a minimum of 22.4 °C to a maximum of 25 °C. The average relative humidity was 78.58% and varied between 70 and 88%. The wind, mainly westerly, blew at an average speed of 5.66 m/s (20.38 km/h), and a maximum gust of 14 m/s (50.4 km/h). S3 was the only soil where its layers were combined prior to lab analysis, because as a control soil, there was no record of sludge application.

### Laboratory analysis

Compost samples were treated as sludge according to Sakali et al. (2021, 2022), based on an H_2_O_2_ protocol, commonly known as wet oxidation peroxide (WPO), followed in sequential time steps to extract the MPs by flotation.

**Fig. 1:**
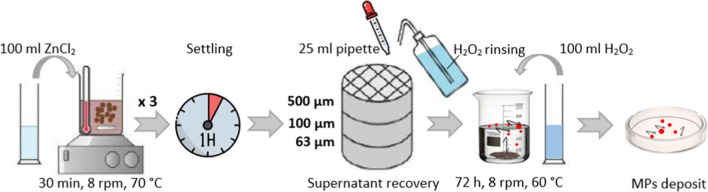
MP separation method from the soil

It has been reported that standard separation methods for MPs from the soil are deficient (Li et al. 2019) and that most are under consideration (Li et al. 2021; Yu & Flury 2021). Therefore, we implemented a methodology based on Zhang et al. (2020) study (Fig. 1), in which the soil samples were pretreated to homogenize and disaggregate, avoiding any agglomeration. Roughly, 5 g of each substrate was processed with ZnCl_2_ hypersaline solution (100 mL, ITW Reagents) on a digital hot plate (Ovan, Spain) at 8 rpm for 30 min. One hour sample settling was required. ZnCl_2_ separation was repeated three times to reach maximal particle range flotation. The supernatants were recuperated with a 25-mL pipette then dropped on three 8-in diameters, 500-, 100-, and 63-µm stainless steel sieves, moved into clean glass beakers. Repeated rinsing of the sieve with H_2_O_2_ (30%) was necessary to recuperate every particle. Thereafter, H_2_O_2_ (100 ml, ITW Reagents) was added to react with final supernatants for 72 h at 60 °C, 8 rpm. The samples were sieved, washed with ultrapure water, and then, filtered through polycarbonate filters (PC) (47 mm ø, 0.8 μm, Isopore™) by a vacuum pump and dried for 2 h at 40 °C to pursue microscopic identification.

A negative control next to each soil and compost sample was included so there would be no compromising the quality of the analysis. The controls enclosed clean, empty beakers that were placed adjacent to the ones containing the actual samples and were processed the same way. All materials employed in the laboratory analysis were made of glass and/or stainless steel.

### Earthworm trials

In order to analyze possible ingestion of MPs by characteristic organism of the agricultural soil fauna (earthworms), an experiment was designed based on the study followed by Baeza et al. (2020). The aim of this experiment was to evaluate if the type of MPs identified in agricultural soils and its average size (ø < 250 μm) could be ingested. Therefore, two types of worms (*Lumbricus terrestris* (LT) and *Eisenia fetida* (EF)) were exposed to different MP types in increasing concentrations (5, 7, and 10%), in reference to previous results showing that higher concentrations affect organisms negatively. However, the selected concentrations in this study could be too high in respect to registered concentrations in natural soils, and therefore, further studies with lower MP concentrations should be followed.

Two tests were carried out, a preliminary test with a substrate conditioned for worms, supplied by a local commercial supplier (Centro de Energía Viva de Andalucía, CEVA), which was previously analyzed in means of MP content according to the exposed methodology for soils, under controlled laboratory conditions (named preliminary trials), and then a second test following the same procedure, but this time with a real soil, containing the highest concentration of MPs (S1) (named agricultural field trial), which was preconditioned with water.

Two polymers that were identified in the sample soils (polyethylene terephthalate (PET) and polypropylene (PP)) in addition to PS (polystyrene) were used. These were, previously ground as bulk shape to the average size identified in this study. For the study, the following plastic materials with known composition, such as plastic straws (PP), plastic spoons (PS), and cork (PET), were used. Sample of about 40 g of each material were crushed to a size larger than 5 mm and then crushed into a much smaller size ranged between 100 and 250 µm with the help of a Retsch Ultra Centrifugal Mill ZM 200, USA (IVAGRO (Instituto de Investigación Vitivinícola y Agroalimentaria)) based on liquid nitrogen. Prior to their use in testing, the materials were analyzed by FTIR to confirm and check the initial materials of the crushed elements, after the first crushing and grinding. In all three cases, the FTIR yielded the same information and confirmed the existence of the reference polymers PET, PP, and PS compared with the commercial polymers available in the laboratory.

Two types of worms, sourced from the same commercial supplier, were investigated *Lumbricus terrestris* (LT) and *Eisenia fetida* (EF). All experiments in this study were followed with adult earthworms (LT 5.1 ± 0.7 g and EF 2.7 ± 0.7 fresh weight). During the period of adaptation to laboratory conditions (approx. 1 month), the earth worms were kept alive by adding crushed apples as food. Prior to the test, the worms were conditioned by emptying their intestine; hence, they were placed on wet filter paper for 24 h. In this way, the worms increased their appetite for soil and encouraged their digging into the soil (Lahive et al. 2022).

**Fig. 2:**
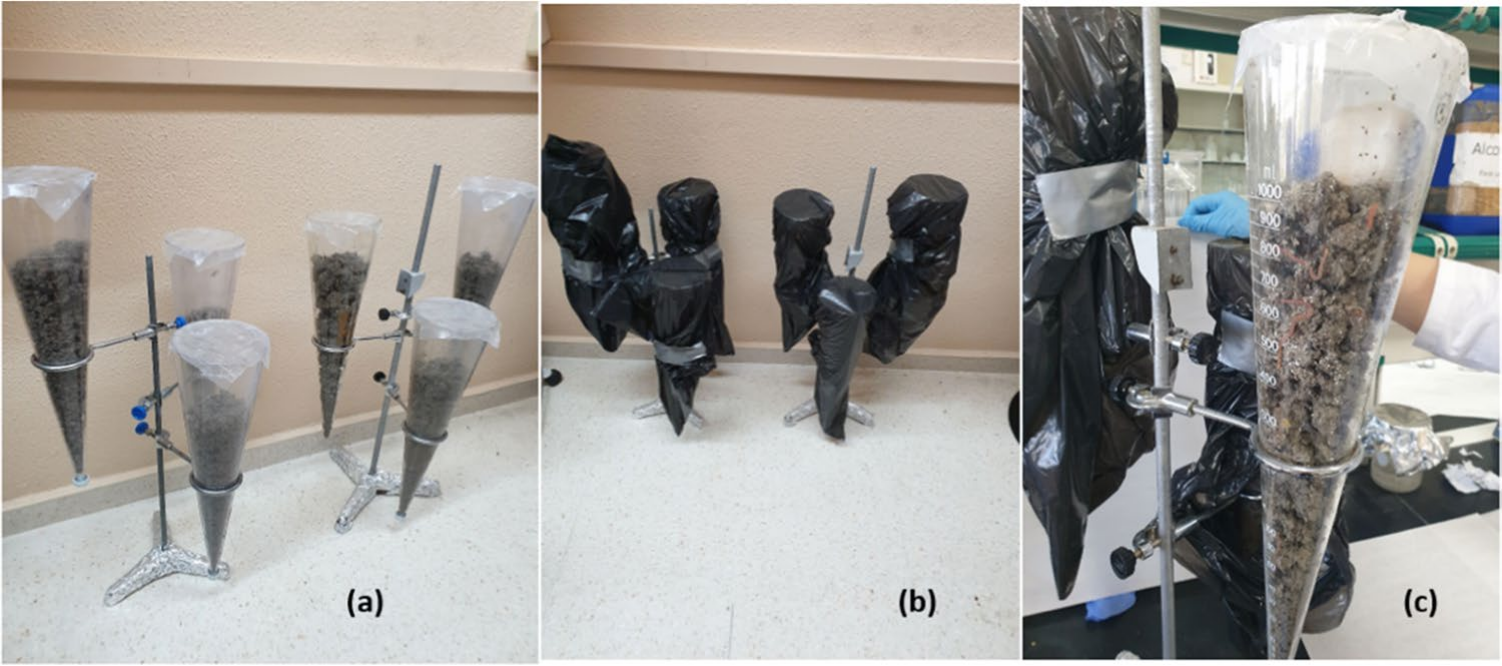
Earthworms’ bioassays: **a** mimic soil conditions, **b** samples in darkness, **c** after MP exposure

For both types of worms, a control (without MPs in soil) in addition to three trials was carried out where the soil was mixed with the MPs in increasing concentrations of 0.9, 2.1, and 3% w/w. For each exposure, four mature worms (with a visible band about 1/3 or less down from the top) were placed. The eight different trials were carried out in glass receptacles (Imhoff cones, India) to maintain a soil column of about 20 cm (Fig. 2) and reproduce similar field conditions in the dark (with the use of rubbish bags).

Table 1 shows the amounts (g) of soil and MP exposures, in addition to the number of individuals (worms) of each type. These trials were maintained for a period of 7 days, after which the worms were individually extracted for subsequent MP analysis according to the protocol established by Zhang et al. (2020), which employs prior digestion of the individuals, followed by a sieving process to retain the microparticles on PC filters.

**Table 1:** MP concentrations in each worm trial

Samples	Soil (g)	MPs (g)			Earthworms (Nº of individuals)	
		PP	PET	PS	LT	EF
LT1 (0.9% w/w)	300	1.5	1.5	1.5	4	4
LT2 (2.1% w/w)	300	3.5	3.5	3.5	4	4
LT3 (3% w/w)	300	5	5	5	4	4
Control 1 (C1)	300	0	0	0	4	4
EF1 (0.9% w/w)	300	1.5	1.5	1.5	4	4
EF2 (2.1% w/w)	300	3.5	3.5	3.5	4	4
EF3 (3% w/w)	300	5	5	5	4	4
Control 2 (C2)	300	0	0	0	4	4

### Instruments and analytical conditions

The filters with the recovered particles were examined by a stereomicroscope (ZEISS Achromat S 1.25 × FWD 50 mm, UK) equipped with a digital ZEISS Axio camera. The observed items were classified as fiber, bulk (granular), or fragment (irregular shape particle) according to their morphologies. The size was measured using the included software, divided into the same range of sieves. The polymer type was identified in total attenuated reflection mode with a Fourier transform infrared (FTIR) spectrometer (Spectrum 100™, PerkinElmer, USA) (Sakali et al. 2021).

### Quality control measure

Monitoring contamination throughout the process is an important fact for the analysis of MPs. Attention should be paid to implementing consistent QA/QC practices from inception through the entire study process (including during the design of the study, sampling and collection process, extraction, and analysis). Cotton clothes and gloves were worn during sampling and analysis to avoid contamination by plastic fibers.

During the experiment, controls of both aerial blanks, soil, and compost samples that formed the substrate for the earthworms were carried out. Mainly acrylic, cellulose, and polyester fibers of 0.2 to 3 mm in length were found. Therefore, these types of polymers were excluded from the results due to a possible source of laboratory contamination.

The data obtained were reported starting with shape, size, type, and abundance of MPs in compost and then soil, ending with an overall comparison between both earthworm species.

## Results and discussion

### Presence of MPs in the compost samples

Fibers and fragments resulted to be the most abundant. Fibers were, on average, 5 times more abundant than fragments. Overall fibers were nonwoven, with much higher elongation, and had an average length of 982.6 µm compared with the overall average fragment of 176.9 µm.

An increase in the number of microparticles during the composting process was also observed, resulting in 286 microparticles in C3 compared with C2, with 193 microparticles and C1 with 157 microparticles.

**Fig. 3:**
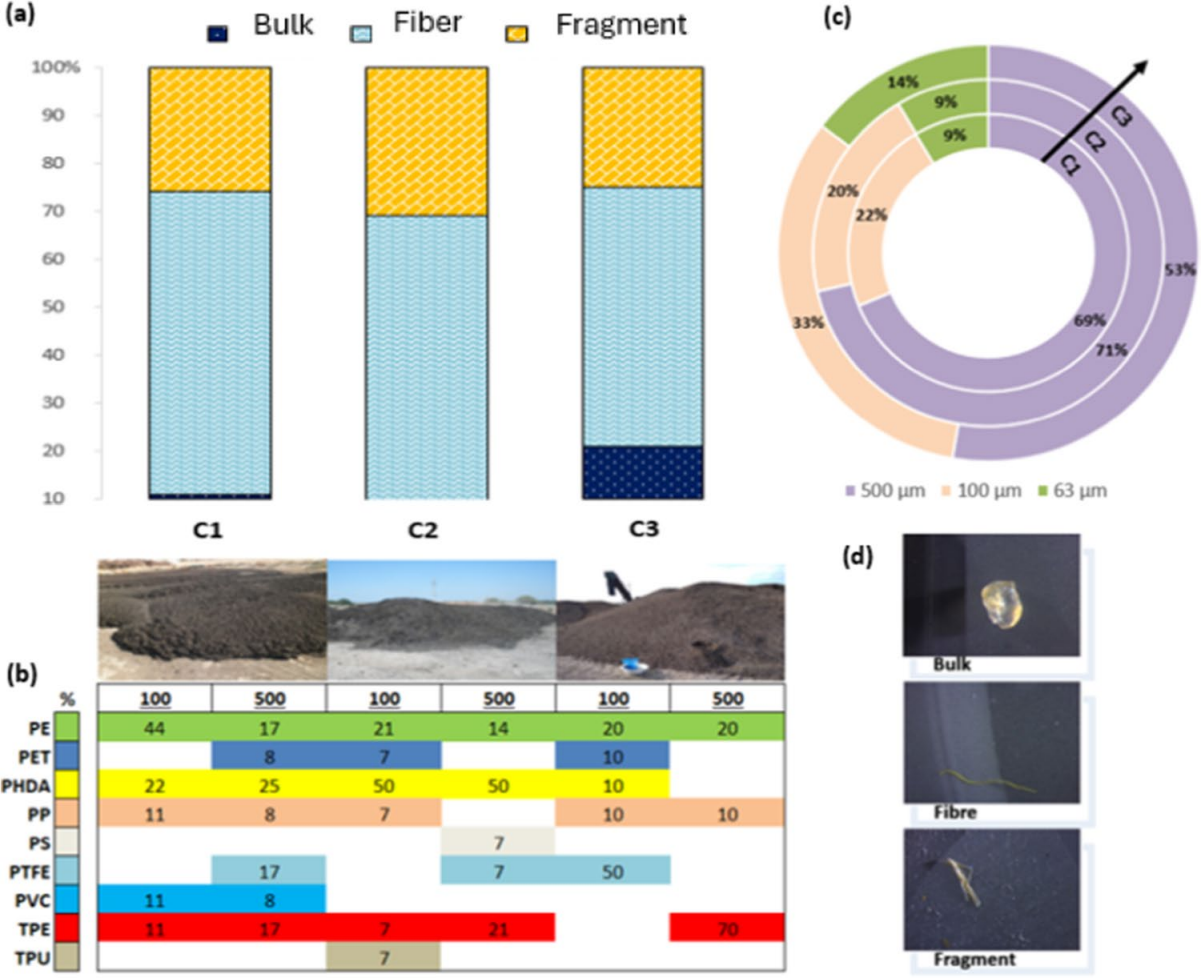
Morphotype (**a**), size distribution (**b**), polymer type (**c**), and photos of MPs shape found in the compost samples (**d**)

In reference to the fibers and fragments presence, in the case of C1, fibers reached an average of 63% and fragments of 26%; in C2, they represented a 60% and 31% respectively; and in C3, the fibers reached 54% and the fragments 25% (Fig. 3a). This is in line with other studies, which mentioned that these are the most abundant in compost (Braun et al. 2020; Edo et al. 2022). The large amount of fibers can be attributed mainly to their release of fibers from the textile industry and the washing of synthetic garments (Henry et al. 2019; Cohen et al. 2021), while in the case of fragments, they usually derive from secondary MPs due to the fragmentation of larger particles (Nguyen et al. 2019). The bulk form was found in smaller proportions (Fig. 3d), with an 11% in C1, a 9% in C2, and 21% in C3 (Fig. 3a).

The behavior of particles during the composting process can be well described by monitoring the evolution of their size. Figure 3b indicates that for all three types of compost, microparticles of 100 µm predominated, especially in C1 and C2. The possible explanation is that small-sized MPs tend to overlap easily due to mass and remain entrapped in the sludge. However, in C3, the difference in microparticle size was smaller. This apparent distinction in the particle size can probably be ascribed to the application amount of sewage sludge and the application history. The highest number of 100 µm microparticles was found in C2 reaching values of up to 150.1 ± 36.1 particles/g, while in C3, the highest number referred to 500 µm microparticles resulting of 141.8 ± 41.8 particles/g corresponding to 71% and 33%, respectively (Fig. 3b).

Curiously, the percentage of particle quantity increased during the composting process, the compost with the highest number of microparticles was the finest with a total of 285.7 ± 30.4 particles/g. This may be due to the fact that, during the composting process, there was some external atmospheric contamination. Therefore, more 500 µm microparticles were found compared with all other types of compost which is similar to the results obtained by EL Hayany et al. (2020). In the case of C3, this could be from mixing C1 with shrub pruning and branches, which is a way plastic becomes incorporated (Jiao et al. 2024).

In this way, although the collected sewage sludge that is used as agricultural amendment and soil compost (Koyuncuoğlu & Erden 2021) is considered to be a valuable and economic mineral product, this is still in question because it is not free of MPs. Via FTIR, an average of 56% of the samples were confirmed as plastic during chemical analysis. Poly (hexadecyl acrylate) (PHDA), polyethylene (PE), polyethylene terephthalate (PET), and polypropylene (PP) were identified among the potentially plastic-derived spectroscopy products; inert polymers were also found, i.e., polymers that hardly react with external agents such as PTFE and others shown in Fig. 3c.

An hypothesis did stand up with respect to the by-products for fermentation of C2, which may have stimulated degradation and loss of organic carbon or added MPs, such as what happened with PHDA.

During the C3 step, PTFE (fragments and fibers) and TPE (overall fibers) demonstrated large increases compared with C2, probably due to the mechanical conditioning and refining of the residue before the sales service.

The MP abundance which attained 171.2 ± 8.3 × 10^3^ MPs/kg (dw) in the final stage of C3 resulted in the same order of magnitude as other studies, which reported that MPs concentrations range from 23.0 ± 5.7 × 10^3^, 26.0 ± 8.3 × 10^3^, and 18.0 ± 3.2 × 10^3^ particles/kg, respectively, in Morocco’s final composts (EL Hayany et al. 2020), 10–30 items/g in northern Spanish dry compost (Edo et al. 2022), and 200 − 420 items/kg with an average of 353.3 ± 97.0 items/kg in Chinese composts (Zhang et al. 2020).

### Presence of MPs in agricultural soils

The average particle distribution (%), within standard errors, was calculated for 28 samples in total. Results showed that all soil samples contained more fibers (from 37% ± 14.8 to 66% ± 11.8 in all layers) than fragments (mean, 28.5% ± 17.7), which results is in line with similar studies (Corradini et al. 2019; Li et al. 2019; Schwinghammer et al. 2020; Crossman et al. 2020; Zhu et al 2021). However, Zhang et al. (2020) only found a fiber dominance in fields that had never plowed nor irrigated with wastewater. In addition, van den Berg et al. (2020) explained the existence of a preference for fragments to be retained in the soil, rather than fibers.

**Fig. 4:**
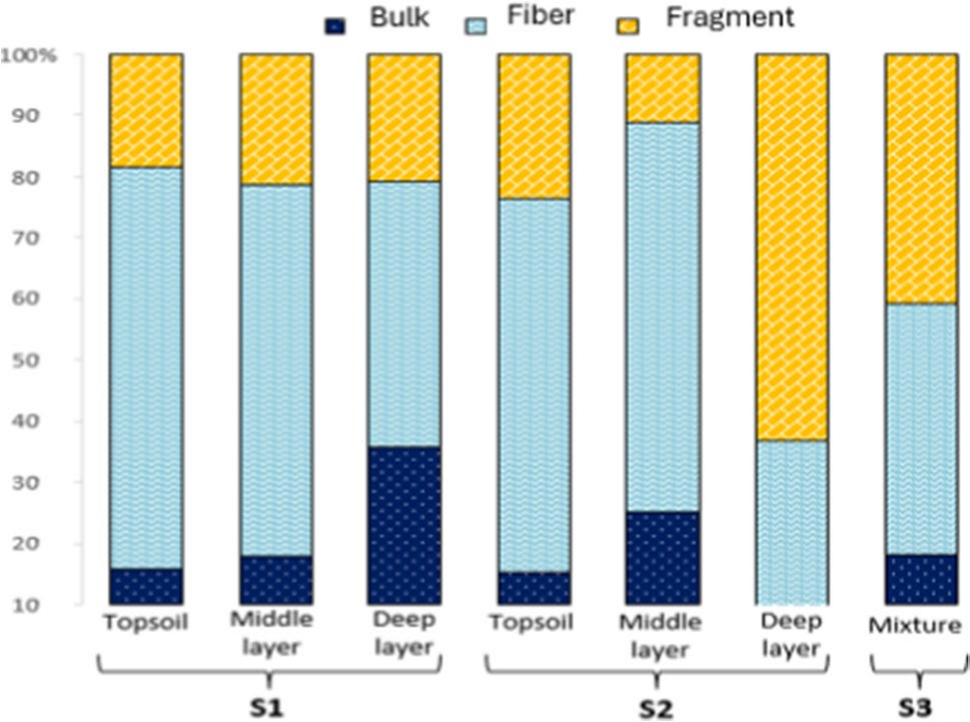
Distribution of particles in the soils (S1, S2, and S3) and depths studied (topsoil (0–5 cm), middle layer (5–10 cm), and deep layer (10–20 cm))

Particle distribution varied significantly, between soils and depths. As shown in Fig. 4 in the case of S1, there was a particle distribution with mean values of 57% ± 11.8 for fibers, 23% ± 11.0 for bulk particles, and 20% ± 1.6 for fragment. Fiber contamination decreased depth unlike that of bulk. It is important to highlight that fragment distribution showed the same particle mobilization in the middle and deep layers. A completely different trend of particle distribution was observed in S2, showing lower particle abundance than in S1; the morphotypes of fiber and bulk had the greatest proportion in the middle layer, while fragments predominantly accounted for 63% ± 27.1 in the deep layer. In all cases, the topsoil was positioned in second place of item retention. The possible explanation is that S2 with the biggest amount of particles, as was expected since it is the soil with more than 4 years of sludge exposure, was pushed towards the bottom (10–20 cm) due to a downward mechanical movement during the soil plowing process which tended to an accumulation in the middle deep layers in contrast to the topsoil (0–5 cm). S3 showed enrichment of fiber and fragment even though there was no record of sludge exposure, apparently; there is previous evidence that atmospheric fallouts can promote their accumulation anywhere (Bianco & Passananti 2020; Evangeliou et al. 2020; Zhang et al. 2020).

**Fig. 5:**
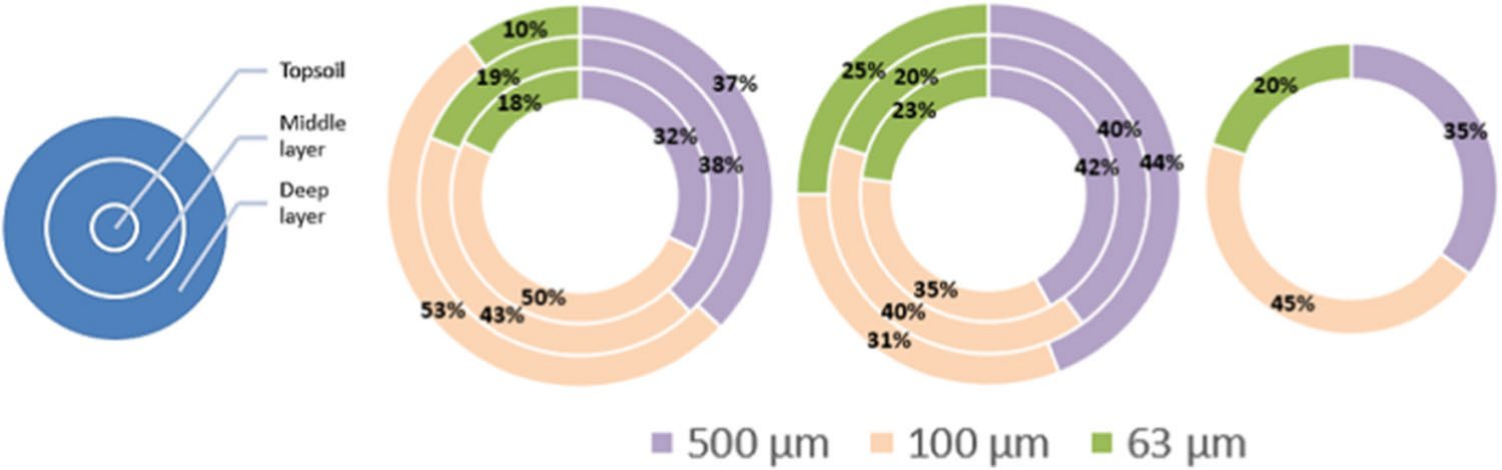
Size distribution in each layer (MPs/g dw) area distribution in the soils (S1, S2, and S3) for the topsoil (0–5 cm), middle layer (5–10 cm), and deep layer (10–20 cm)

For the three soils studied, the size distribution in Fig. 5 shows that nearly two-fifths (42.5% ± 7.8) of the particles were larger than 500 µm and only 19.3% ± 4.8 were bigger than 63 µm on average in all soil layers, which is in line with previous studies (Yu et al. 2021; van Schothorst et al. 2021; Zhang et al. 2020). Also, particles bigger than 500 µm were also abundant in the topsoil and account for 50% of S1. S3 demonstrated a significant net of particles (> 500 µm) equal to 45%. In addition, medium-sized particles (> 100 µm) had a remarkably higher percentage (42%) in the topsoil layer than bigger particles (35%) in the case of S2. Size distribution has also been scrutinized in other urban settlements and was dominated by < 50 µm sized particles in India (Sarkar et al. 2022) and < 250 µm in the Netherlands (Cohen et al. 2021). This difference in size composition implies that some particles may have been decaying in nature for extended periods of time. Also, the sludge can transport suspicious particles into the soil that filter their toxic effects in depth (Milojevic & Cydzik‐kwiatkowska 2021).

No significant depth-dependent differences in particle size were observed within the other two layers left in S1. In this way, particles bigger than 500 µm remained similar in all layers. In the case of S2, the 100 and 500 µm particles matched results in the middle layer, achieving a 40%, showing a tendency to dominance of larger particles in the deep layer (44%). The distribution of particles smaller than 63 µm in S1 was as follows: 18% in the topsoil, 19% in the middle layer, and 10% in the deep. In the case of S2, these particles resulted predominant and accounted for 25% in the deep layer showing a slightly drop off with decreasing depth, while S3 scored 20% even without plowing.

The achieved results revealed that the particle size distribution in both S1 and S3 was distorted; in contrast to Yu et al. (2021), only the deepest layer in S2 showed a retention preference for 63 µm < particles < 100 µm with high percentages, which may be related to soil density, surface runoff, recent plowing, or fauna burrows making (Yu & Ma 2020).

**Fig. 6:**
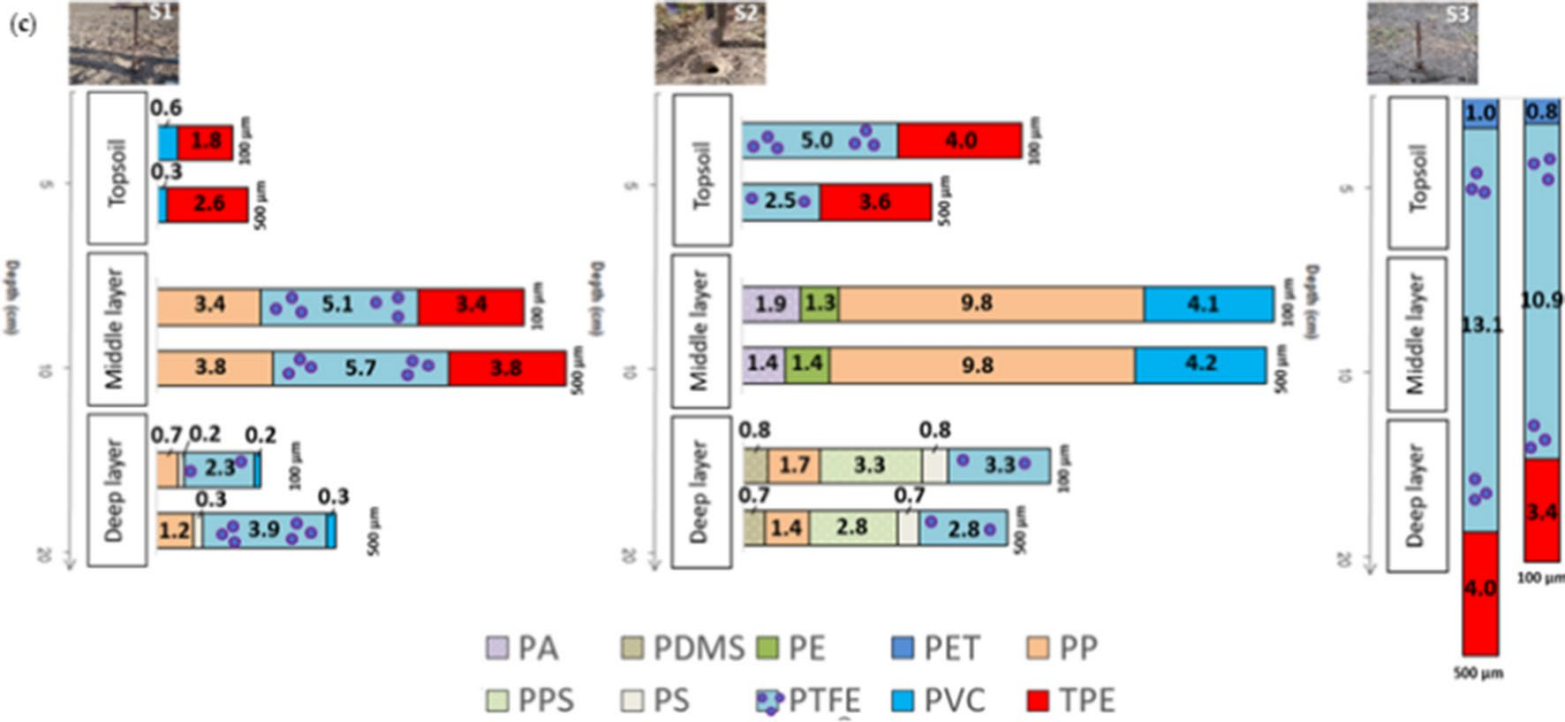
Type of MPs in each layer (MPs/g dw)

Chemical identification of microparticles by FTIR confirmed that a mean, 45%, are indeed MPs. Soil samples showed a wide variety of them. Figure 6 shows the number of MPs per gram obtained for each soil sample according to size fractions. Expressing size distribution this way, instead of by percentage, shows slight difference depending on of 100 and 500 µm, which was not appreciated when displayed as percentage unity.

PTFE dominance in the topsoil could be owed to the fact that it is an inert polymer originated from the wash of daily Teflon utensils, while TPE dominance can be related to car tyres or building insulation. In fact, the most abundant polymer, overlapped in all layers, is PTFE, which scores 54.8 MPs/g dw. This polymer has many applications such as thread for sewing products that are continuously exposed to atmospheric and/or chemical agents. It is also used in agricultural irrigation systems to prevent water leaks in pipes or stopcocks, and it is found in textile products (Seymour and Carraher 2002). The second most abundant polymer in the soil samples was PP (31.8 MPs/g dw), probably because of its many industrial applications including electrical and electronic components, industrial materials, vehicle parts and components, packaging, cosmetics, and medicines (Seymour and Carraher 2002; Mukherjee et al. 2023). In addition, other studies also showed that these polymers appeared with an average abundance of approximately 15% (Bayo et al. 2020). In our study, the percentages are somewhat different (Fig. 6). PTFE corresponds to 34.9% of the MPs found and PP to 20.2%, which is not in similitude with the MPs% transfer from biosolids to the soils of agricultural fields to which they were applied, in Ontario, Canada (Crossman et al. 2020).

Therefore, the results obtained in this study showed that soil subjected to higher doses of fertilization presented a higher abundance of MPs. Resulting in 58.8 × 10^3^ MPs/kg for S2 (with the lowest dose at 15t/ha) versus 84.0 × 10^3^ MPs/kg for S1 (the highest dose at 30 t/ha), minor abundance was registered by S3 (33.3 × 10^3^ MPs/kg). When comparing data with literature, these exceeded those of Chile and Canada with an average of 34 × 10^3^ MP particles/kg (Corradini et al. 2019) and 11.5 × 10^3^ MPs/kg (Crossman et al. 2020), respectively; and wat above those found in China with an average of 316.75 elements/kg (Zhang et al. 2020). However, they resulted similar to those found in northern Spain with an average of 50 × 10^3^ MP particles/Kg (van den Berg et al. 2020).

### MPs ingestion assays in earthworms

After 7 days of exposure, the worms were individually extracted from the soil. Once pulled out they were weighed and measured, after being thoroughly rinsed and wiped carefully, showing no differences in weight or length, and all with healthy appearance.

**Fig. 7:**
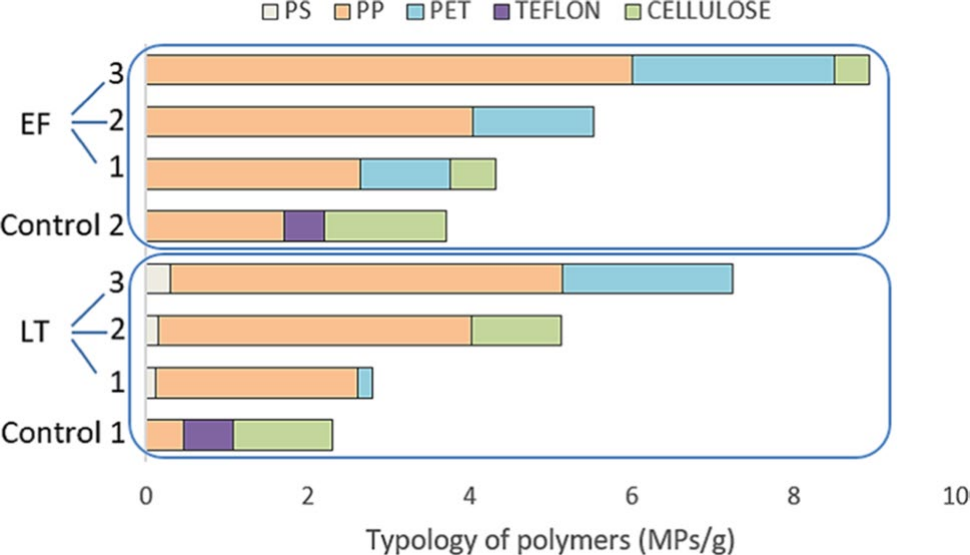
Typology of polymers found in the examined species

Regarding the identification and quantification of the types of polymers ingested, both species showed a progressive increase in the total content with a rise of exposure to MPs. Hence, for *Lumbricus terrestris*, results were of 2.79 MPs/g body weight for LT1 compared with 5.12 MPs/g of body weight in LT2 and 7.25 MPs/g of body weight in LT3, while the control, C1, resulted with 2.3 MPs/g body weight. Independent from the polymers dosed, the LT worm ingested PS in all 3 trials, although in a very small amount, and two of the trials showed the presence of PET. It was observed that in the case of PP, the control worms had already ingested this polymer, possibly because the soil used for the preliminary tests contained it prior to testing, stating an increase with the dose, being this polymer the most easily incorporated during their ingestion (Fig. 7).

The same behavior was registered for *Eisenia fetida* as the content of microplastics per body weight increased with the added dose (4.31, 5.53, and 8.93 MPs/g), for EF1, EF2, and EF3, respectively. Moreover, this invertebrate showed more appetite for PP, followed by PET; however, there was no evidence of PS ingestion in any of the trials (Fig. 7). In both controls, the earthworms ingested PP and cellulose, although the latter was not identified in the experimental soils (Fig. 7). Also, the EF species, despite being smaller, ingested more microparticles per gram than the larger ones. There is previous evidence of the voracity of *Eisenia fetida* in addition to a high perforation power (Chen et al. 2020).

In the case of S1, the soil with the highest contamination by MPs, due to its silty-clayey to clayey texture a preparation with water was necessary before use; therefore, the water retention capacity (WHC) was analyzed (45 ml per 100 g of soil). However, during the acclimatization period both species of worms ended up dying within 7 days, observing rigidity and dryness of the worms. This could be due to various causes, such as soil acidity due to excess of nitrogenous or high protein substances, excess moisture, or even overly dry substrates (Bueno 2015).

A study on the global distribution of earthworm diversity (Phillips et al. 2019) found that climatic variables and habitat cover are more important in shaping earthworm communities than soil properties; however, another recent study on the state of soil degradation (Rodríguez-Berbel et al. 2022) considers contamination by microplastics as one of the main problems, along with overgrazing, salinization, and the use of agrochemicals.

According to this latest investigation, our data corroborate the presence of MPs in agricultural soils amended with compost from sewage treatment plants (with a common size of MPs of < 250 µm in diameter), and this study also confirms that earthworms are capable of ingesting them. As it can be seen in Fig. 7, results show that a higher dose of MPs/g available in the environment result in a higher ingestion. Therefore, the doses 3% w/w was the one that showed the highest concentration of MP/g in the individuals for both species of earthworms studied.

## Conclusions

The study of MP inputs into agricultural soils originating from applied commercial composts or sewage sludge, together with the study of the sequential update by soil biota is of great importance to understand the fate and transport of MPs in the environment.

The amount of MPs found in the fine compost samples (S3, control) were 33.3 × 10^3^ MPs/kg. While in the case of the sludge-enriched soil, MPs ranged from 58.8 × 10^3^ MPs/kg (S2, the lowest dose at 15t/ha) to 84.0 × 10^3^ MPs/kg (S1, the highest dose at 30 t/ha).

This study identified compost and sludge as a major solid source of MPs. Moreover, there is a clear increase in the relationship between the stage in the composting process and the numbers of particles and MPs increase. Both plowed soils studied demonstrated an abundance of MPs in the middle layer supporting the hypothesis that the abundance and particle size of MPs (63–100 µm) in agricultural soils increase with increasing fertilization and composting stage.

Regarding the micropollutant transition to the worms embedded in cones, there was hardly any influence observed with respect to the MP dosage in the first trials for both species studied (LT1 and EF1). However, we could confirm that the lower the dose of MPs/g available in the environment, the lower the ingestion. Therefore, the amount of MPs/g detected in both species of earthworms was studied, being the dose of 3% w/w the one that shows the highest concentration of MPs/g in the individuals for each species.

We could then consider that from this concentration of MPs in agricultural soils, and of the most identified size (diameter < 250 µm), a considerable intake by these annelids could occur. Regarding the type of MP ingested, PP was found in a higher proportion. When comparing both species, it turned out that *Eisenia fetida* showed a greater voracity than *Lumbricus terrestris*, due to its higher content of microparticles per gram of body weight.

Results in this study disclose the recommendation of the development of further studies that can reveal threshold data of MPs in agricultural soils, in order to establish good practices in technical standards for fertilization. In addition, there should be more research in the incorporation of MPs in the case of reused, especially due to its role to promote the principles of the circular economy. Last, but not least, MPs intake is of major importance in order to be able to establish a level of toxicity for the standard organisms that inhabit agricultural soils.

## Data Availability

The authors declare that data supporting the finding of this study are available within the paper. If raw data files in another format are required, they are available from the corresponding author upon reasonable request.

## Nomenclatures

EF: *Eisenia fetida*
EPS: Extracellular polymeric substances
FTIR: Infrared spectroscopy
H_2_O_2_: Hydrogen peroxide
MPs: Microplastics
LT: *Lumbricus terrestris*
PAHs: Polycyclic aromatic hydrocarbons
PCBs: Polychlorinated biphenyls
PC: Polycarbonate
PE: Polyethylene
PET: Polyethylene terephthalate
PHDA: Poly (hexadecyl acrylate)
PP: Polypropylene
PS: Polystyrene
PTEs: Potentially toxic elements
PTFE: Polytetrafluoroethylene
PVC: Vinyl polychloride
SDGs: Sustainable Development Goals
TPE: Thermoplastic elastomers
WHC: Water retention capacity
WPO: Wet oxidation peroxide
WWTP: Wastewater treatment plants
